# Successful malaria elimination strategies require interventions that target changing vector behaviours

**DOI:** 10.1186/1475-2875-12-56

**Published:** 2013-02-07

**Authors:** Tanya L Russell, Nigel W Beebe, Robert D Cooper, Neil F Lobo, Thomas R Burkot

**Affiliations:** 1 James Cook University, Queensland Tropical Health Alliance, Cairns, Queensland, Australia; 2 University of Queensland, School of Biology, St Lucia, Queensland, Australia; 3 CSIRO Ecosystem Sciences, Dutton Park, Queensland, Australia; 4 Australian Army Malaria Institute, Gallipoli Barracks, Enoggera, Queensland, Australia; 5 Department of Biological Sciences, University of Notre Dame, Eck Institute for Global Health, Indiana, USA

## Abstract

**Background:**

The ultimate long-term goal of malaria eradication was recently placed back onto the global health agenda. When planning for this goal, it is important to remember why the original Global Malaria Eradication Programme (GMEP), conducted with DDT-based indoor residual spraying (IRS), did not achieve its goals. One of the technical reasons for the failure to eliminate malaria was over reliance on a single intervention and subsequently the mosquito vectors developed behavioural resistance so that they did not come into physical contact with the insecticide.

**Hypothesis and how to test it:**

Currently, there remains a monolithic reliance on indoor vector control. It is hypothesized that an outcome of long-term, widespread control is that vector populations will change over time, either in the form of physiological resistance, changes in the relative species composition or behavioural resistance. The potential for, and consequences of, behavioural resistance was explored by reviewing the literature regarding vector behaviour in the southwest Pacific.

**Discussion:**

Here, two of the primary vectors that were highly endophagic, *Anopheles punctulatus* and *Anopheles koliensis*, virtually disappeared from large areas where DDT was sprayed. However, high levels of transmission have been maintained by *Anopheles farauti,* which altered its behaviour to blood-feed early in the evening and outdoors and, thereby, avoiding exposure to the insecticides used in IRS. This example indicates that the efficacy of programmes relying on indoor vector control (IRS and long-lasting, insecticide-treated nets [LLINs]) will be significantly reduced if the vectors change their behaviour to avoid entering houses.

**Conclusions:**

Behavioural resistance is less frequently seen compared with physiological resistance (where the mosquito contacts the insecticide but is not killed), but is potentially more challenging to control programmes because the intervention effectiveness cannot be restored by rotating the insecticide to one with a different mode of action. The scientific community needs to urgently develop systematic methods for monitoring behavioural resistance and then to work in collaboration with vector control programmes to implement monitoring in sentinel sites. In situations where behavioural resistance is detected, there will be a need to target other bionomic vulnerabilities that may exist in the larval stages, during mating, sugar feeding or another aspect of the life cycle of the vector to continue the drive towards elimination.

## Background

Following the discovery by Ronald Ross that mosquitoes transmit malaria, attempts to control the disease focused on attacking the vector mosquitoes. These early attempts to control malaria focused on the larval stages (which were easier to find than the mobile adults) using environmental management and larviciding. This approach was difficult to implement uniformly and effectively across wide geographic areas as vectors can use a diverse and extensive range of breeding sites that vary by species. The challenge is compounded by the fact that there are at least 41 dominant vectors and possibly scores more secondary vectors that transmit malaria [1]. When studies revealed that host-seeking female mosquitoes enter houses to feed on sleeping people and then rest inside the house after feeding; this stage of the mosquito life cycle was targeted for control using insecticides. In the 1930s, indoor spraying was successful in India and South Africa using pyrethrum; but the lack of residual activity meant that this compound had to be sprayed weekly and therefore was logistically and financially challenging to implement [2,3]. This limitation was overcome in 1939 when the residual insecticidal activity of DDT was discovered, making large-scale indoor residual spraying (IRS) operations financially viable. During World War II, the US Army controlled malaria outbreaks among troops deployed in the southwest Pacific using DDT-based IRS (DDT-IRS) [4]. The success of this method spawned numerous field trials in the 1940s-50s throughout the Americas, Africa, Asia and the Pacific. The impressive results in controlling malaria across this wide geographic range suggested that malaria eradication could be feasible using DDT-IRS.

### Malaria eradication efforts: history revisited

In 1955, the World Health Organization (WHO) launched the Global Malaria Eradication Programme (GMEP) based primarily on DDT-IRS supplemented with mass drug administration in malaria-endemic countries outside of sub-Saharan Africa [5]. The GMEP was moderately successful, eliminating malaria from 37 countries; however it was unable to eradicate malaria. There were a number of technical, administrative, financial, and logistical challenges that contributed to this disappointing outcome.

One of the technical reasons for the failure to eliminate malaria was over reliance on a single intervention and the subsequent development of behavioural resistance by the mosquito vectors [6-8]. DDT was shown in some areas to irritate mosquitoes and reduce both the rate of house entering and successful blood feeding by those mosquitoes that did enter the house [9,10]. Those mosquito populations that responded by changing their behaviour to avoid DDT by feeding outdoors and not resting indoors had a selective advantage. This was observed after DDT use in various species including *Anopheles farauti*[9,10], *Anopheles sundaicus*[11]*, Anopheles pseudopunctipennis*[12,13] and *Anopheles albimanus*[12-14]. Towards the end of the GMEP, transmission was being maintained in many “problem areas” by physiologically susceptible vectors that avoided or minimized their exposure to DDT [6-8].

With the collapse of the GMEP, malaria cases steadily increased. Faced with pandemic transmission levels, the global interest in alternative vector control tools was renewed leading to the development of insecticide-treated nets (ITNs) and then wash-resistant, long-lasting insecticide-treated nets (LLINs). LLINs, like IRS, kill vectors inside houses but also provide a physical barrier against mosquitoes attempting to feed on sleeping humans. During the past decade, LLIN use increased across the malaria-endemic world alongside improved treatment of infected individuals with artemisinin combination therapy (ACT). These measures significantly reduced the global malaria incidence and led the Bill & Melinda Gates Foundation (BMGF) and the WHO to announce that global malaria eradication was again a public health priority. This goal was subsequently endorsed by the Roll Back Malaria programme in 2007. Similar to the first GMEP, the present-day strategies include vector control inside houses (LLINs and IRS) as a critical element.

The problems faced during the original GMEP highlight that programme success will require vector control interventions to target species-specific vector behaviour. Notably, under insecticide pressure vector populations can change over time in several ways: 1) physiological resistance to insecticides can develop in which mosquitoes exposed to insecticides are not killed, and/or, 2) the relative composition or abundance of multiple vectors in an area can change, and/or, 3) the behaviour(s) of individual mosquito species can change. Of these phenomena, physiological resistance is the most straightforward and easy population change to detect and has been widely documented [15], and its monitoring has been implemented into control strategies.

In areas with sympatric vector species where IRS or LLINs are used, those that do not enter houses will have a selective survival advantage over vectors that do enter houses, due to the latter’s exposure to the insecticides in LLINs and IRS. This will result in a selective decline in the population of house-entering and indoor-feeding (endophagic) species relative to more outdoor-feeding (exophagic) species. As such, effective control of the endophagic mosquitoes with IRS or LLINs may not eliminate transmission, as exophagic vectors in the area (which may have been previously considered secondary in importance) may maintain transmission [16]. Recent examples of such scenarios were reported for the *Anopheles gambiae* complex in Kenya, Tanzania and Equatorial Guinea. Here, insecticidal pressure selectively reduced the density of the endophagic and physiological susceptible *An. gambiae s.s.*, while the density of the exophagic sibling species *Anopheles arabiensis* has remained relatively stable. Resultantly, the level of transmission vectored by the entire complex has reduced, but the proportion of the residual transmission which occurs outdoors has increased [17-20].

Less frequently reported, but no less a threat to effective malaria vector control, are changes in the behavioural phenotypes expressed within individual species after implementation of LLINs and IRS. While the mechanisms underlying phenotypic evolution aren’t clearly understood, this phenomenon can occur under extreme selection pressure. Generally, individuals can modify their behaviour in response to stressful events, and these selected traits are then inherited onto sequential generations. This natural selection leads to the eventual transformation of the population to contain the selected trait [21]. Because of the relatively short generation time of mosquitoes, genetic shifts in behaviour in response to selective pressures induced by interventions may be expressed rapidly. Such behavioural resistance has been documented for *Anopheles farauti* in the southwest Pacific [22]*.*

### A tale of three mosquito species

In the southwest Pacific there are three primary malaria vectors, *An. farauti, Anopheles koliensis* and *Anopheles punctulatus. Anopheles punctulatus* and *An*. *koliensis* are endophagic species feeding late in the night (predominant biting activity occurring around midnight). *Anopheles farauti* feeds throughout the night but commences feeding, in relatively high densities, early in the evening (6.30 pm) [22-24].

In this region, DDT-IRS, implemented during the 1960s and 1970s, initially controlled all three species; but the sustained impact of this tool depended on the vectors to continue entering houses. After repeated spray rounds, the highly endophagic *An. punctulatus* and *An. koliensis* became virtually impossible to find [22,25-27]; though the long-term duration of the impacts ranged from transient suppression in Indonesian Papua to prolonged population reductions in Papua New Guinea to continuing scarcity after cessation of IRS in the Solomon Islands where the most intensive operations were implemented [9,22,28]. In fact, *An. koliensis* is thought to have been eliminated from the Solomon Islands. The populations of *An. punctulatus* and *An. koliensis* expressed little plasticity in their feeding behaviour and these populations did not develop behavioural resistance.

The IRS programme became ineffective in many areas when *An*. *farauti* populations quickly rebounded to pre-spray levels within a few years despite continual spraying pressure [22,27]. Populations of *An. farauti* were able to recover by changing their biting behaviour to seek blood meals outdoors and early in the evening before people were protected by the intervention. Prior to DDT-IRS use, the percentage of *An*. *farauti* biting before 9 pm ranged from 11 to 40% with equal feeding indoors and outdoors [10,22-24]. When DDT-IRS was implemented, the percentage of biting before 9 pm rapidly increased to more than 70% and the majority of biting shifted to outdoors [9,10,22,27]. This behavioural change was documented at multiple locations in Indonesian Papua [9], Papua New Guinea [27,28], the Solomon Islands [22] and Vanuatu [10]. After the DDT pressure was eventually removed from the mosquito populations, the modified behaviour persisted. Treated bed nets elicited a similar selection pressure as IRS and behavioural changes in *An*. *farauti* were documented after ITNs were introduced [29]. Thus the protective efficacy of both IRS and LLINs is limited by mosquitoes which circumvented exposure to the interventions through behavioural insecticide resistance, as documented by changes in the vector bionomic traits of time and location of feeding with continuing malaria transmission.

The behavioural changes seen in *An*. *farauti* populations resulted from a change in the behaviour of a single panmictic species which originally expressed very plastic blood meal-seeking behaviours; and not from two behaviourally distinct cryptic species (as seen with *An. gambiae s.s.* and *Anopheles arabiensis* in Africa) with insecticide pressure driving a change in species composition by eliminating the more late night and indoor feeding species [28]. Reasons refuting the latter hypothesis are based on analyses of *An*. *farauti* by DNA-based methods from unsprayed areas, and from areas well outside the flight range of human habitation and which have all confirmed the presence of a only single species [30]. Furthermore, in areas of Papua New Guinea where insecticidal pressure was minimal, molecular analysis of *An*. *farauti* populations that retained the classical pre-spray, night-biting pattern failed to reveal the presence of any late-night biting cryptic species [29,30]. Thus the observations represent true behavioural changes within *An*. *farauti* in response to selective pressure to avoid insecticides. This distinction of a behavioural trait being selected within one species opposed to the selective suppression of one cryptic species has important implications for malaria vector control programmes. Especially because behavioural changes within one species can lead directly to programme failure and rebounds in transmission, whereas the suppression of a dominant cryptic species may simply slow the future progress of vector control programmes.

### Lessons to be learned

There are lessons to be learned from *An*. *farauti* in the southwest Pacific that have global implications for the ability of malaria vectors to circumvent interventions. Throughout the malarious tropics there is a monolithic reliance on indoor vector control with IRS and LLINs and an almost inevitable outcome of effective long-term, widespread use of these tools is that vector populations will change over time, either in the form of physiological resistance, changes in the relative species composition or behavioural resistance. Changes in vector species compositions and changes in behaviour within a species are less frequently seen compared with physiological resistance – possibly because physiological resistance is relatively simple to measure. However, behavioural resistance is potentially more challenging to control programmes. In scenarios where dominant vectors diminish in abundance, the challenge exists that if insecticide pressure is withdrawn the dominant species may reinvade or increase in abundance from vector populations outside the intervention area. Hence, intense vector control must be maintained to prevent the re-emergence of the formerly dominant vector and supplemental additional measures must be implemented to control transmission by the remaining and formerly secondary but now relatively more important species. In the scenario of the species that responded with a change in behaviour, the lesson to be learnt is that the intervention has lost its effectiveness and additional measures will certainly be needed to maintain the same level of effective control.

It is essential that the malaria community acknowledges the very real threat posed by behavioural resistance to the future progress of vector control programmes. The scientific community needs to urgently develop systematic methods for monitoring behavioural resistance and then to work in collaboration with vector control programmes to implement monitoring in strategic sentinel sites. Such monitoring will involve estimating key vector behaviours based on the mode of action of the interventions being implemented. For example, where LLINs are used, the indoor and outdoor biting rate of each species throughout the night in the same location over time should be monitored; in IRS areas, monitoring of resting behaviour is necessary to detect changes in response to the interventions. In situations where a change in species composition or behavioural resistance arises there will be a need to respond with appropriate interventions that target vulnerabilities in the vectors’ behaviours. A loss in intervention effectiveness as manifested by changes in vector species compositions and behavioural resistance cannot be solved by maintaining indoor interventions with alternative insecticidal modes of action. Therefore, there is a need to target other bionomic vulnerabilities that may exist in the larval stages, during mating, sugar feeding or another aspect of the life cycle of the vector. Complementary tools that target such vulnerabilities and further suppress malaria transmission by providing personal protection or reducing the survival, fitness and transmission potential of vector populations will be essential to continue the drive towards elimination. Many of these complementary tools remain at the proof of principle stage and there is a need to prioritize research funding to facilitate village-scale case-controlled investigations across different ecosystems with varying levels of malaria endemicity. Also, future programmatic implementation of vector control will need to be flexible and responsive to changing vector behaviours.

## Competing interests

The authors declare that they have no competing interests.

## Authors’ contributions

All authors contributed to the original idea and writing of the manuscript. All authors read and approved the final manuscript.
